# Effects of autophagy inhibitor 3-Methyladenine on ischemic stroke: A protocol for systematic review and meta-analysis

**DOI:** 10.1097/MD.0000000000023873

**Published:** 2021-01-29

**Authors:** Ao Zhang, Yangyang Song, Zhihui Zhang, Siyuan Jiang, Siqi Chang, Zhengyun Cai, Furong Liu, Xinchang Zhang, Guangxia Ni

**Affiliations:** Nanjing University of Chinese Medicine, Nanjing, China.

**Keywords:** 3-MA, autophagy, infarct volume, ischemic stroke, meta-analysis, neuro-behavioral score, systematic review

## Abstract

**Background:**

Ischemic stroke is a huge threat to human health globally. Rescuing neurons in the ischemic penumbra (IP) is pivotal after the onset of ischemic stroke, and autophagy is essential to the survival of IP neurons and the development of related pathological processes. As the most common autophagy inhibitor, 3-Methyladenine (3-MA) is widely used in studies related to the mechanism of neuronal autophagy in ischemic stroke; however, there is no consensus has been reached on its effects of neuroprotection or neurodamage, which hinders the development and clinical application of autophagy-targeted therapy strategies for the treatment of ischemic stroke.

**Methods:**

We will search the following electronic bibliographic databases: PubMed, EMBASE, Scopus, Science Direct, and Web of Science. Participant intervention comparator outcomes of this study are as flowing: P, animal models of ischemic stroke; I, received 3-MA treatment merely; C, received only vehicle or sham treatment, or no treatment; O, Primary outcomes are infarct volume; neuro-behavioral scores. Secondary outcomes are cerebral blood flow, blood–brain barrier permeability, cerebral hemorrhage, brain water content. Review Manager 5.3 and Stata 15.1 will be used in data analysis. The characteristics of the studies, the experimental model, and the main results will be described, the quality assessment and the risk of bias assessment will be conducted. A narrative synthesis will be made for the included studies. Besides, if sufficient qualitative data is available, a meta-analysis will be conducted. *I*^2^ statistics will be used to assess heterogeneity.

**Discussion:**

This systematic review and meta-analysis of the autophagy inhibitor 3-MAs effects on animal models of ischemic stroke can help us to understand whether inhibiting autophagy brings protection or damage to IP neurons; in addition, it also helps to clarify the specific role of autophagy in cerebral infarction. Therefore, this study can provide evidence for the future development of therapy strategies targeting autophagy and bring more hope to patients with ischemic stroke.

**PROSPERO registration number:**

CRD42020194262.

## 1 Introduction

Ischemic stroke (also known as cerebral infarction), accounts for around 80% of all strokes, which is a leading cause of disability and the second highest cause of human death worldwide.^[1]^ The incidence of ischemic stroke has been increasing in many countries, which poses a great threat to human health; the pathological mechanism and treatment methods of ischemic stroke have become the main focus of stroke research.^[2]^

Right after the onset of ischemic stroke, there occurs the irreversible necrosis in the center of cerebral ischemic infarction.^[3]^ However, due to the collateral circulation, neural death in the area around the infarct center is relatively delayed, and the further process of damage and death of neurons in this area can still be reversed, so these neurons can possibly survive and restore their functions; this area is called ischemic penumbra (IP).^[4]^ Therefore, to rescue the neural tissue of IP area, the primary goal of treating ischemic stroke is to reverse the death process of IP neurons.^[5]^

In response to ischemic brain injury stress, IP neurons initiate the reversible programmed death process; autophagy, as Type II programmed cell death, is the key mechanism of the whole process.^[6]^ Under the stress conditions of ischemia, hypoxia and nutritional deficiency caused by ischemic stroke, autophagy process of IP neurons is activated: autophagosomes are produced to wrap damaged organelles, misfolded macromolecules, and long-lived proteins etc., and then combine with lysosomes and degrade.^[7]^ Accumulating evidence proves that autophagy plays an irreplaceable role among the many factors that determine the fate of IP neurons; autophagy is pivotal to the survival of IP neurons and therefore affects the occurrence and development of related neurological symptoms in ischemic stroke^[8,9]^; scientists have found that autophagy is an important target for the treatment of ischemic stroke.^[10]^

However, autophagy is often described as a double-edged sword in the regulation of cell death in ischemic stroke^[11]^: many studies show that autophagy causes excessive degradation of cellular contents, which leads to cell death, and ultimately damages tissues and organs^[12–14]^; meanwhile, other evidence demonstrates that autophagy helps neurons in degrading and reusing materials, so the basic physiological activities of neurons can be maintained, and the repairment can be promoted, which is conducive to IP neurons survival.^[15–17]^ The specific effect of autophagy on IP neurons remains controversial so far, which encumbers the research on autophagy-targeted therapy strategies, though multiple experiments on animal models with ischemic stroke have proved that a variety of neuroprotective strategies are related to the regulation of neuronal autophagy.^[18–20]^

As the most commonly used autophagy inhibitor drug, 3-Methyladenine (3-MA) is widely used in animal experiments related to the mechanism of neuronal autophagy in ischemic stroke and has shown its significant neurological impacts on animal models of cerebral infarction.^[21–23]^ However, it has not yet been determined whether 3-MA can protect or damage neurons after cerebral infarction by inhibiting autophagy, which not only hinders its further development in research and prevents its clinical application on patients with ischemic stroke, but also brings more difficulties in clarifying the specific role of autophagy.

Therefore, it is necessary to conduct a systematic review and meta-analysis based on the most comprehensive and latest resources to answer the question of “is the autophagy inhibitor 3-MA neurologically beneficial or harmful to animal models of cerebral ischemia?”, which is meaningful to the development of therapy strategies targeting autophagy in the treatment of ischemic stroke.

## 2 Methods

### 2.1 Study registration

This systematic review protocol has been registered on PROSPERO with number CRD42020194262 (https://www.crd.york.ac.uk/PROSPERO/display_record.php?RecordID=194262).

The protocol report was conducted according to the Preferred Reporting Items for Systematic Review and Meta-analysis Protocol Guidelines (PRISMA-P)^[24]^ and the recommendations for reporting systematic reviews and meta-analyses of animal experiments.^[25–27]^ Any change of the review will be described if needed. This systematic review and meta-analysis will be published in a peer-reviewed journal. Formal ethical approval and informed consent are not required for this study, because this study is a secondary research based on previously published studies.

### 2.2 Inclusion criteria

#### 2.2.1 Types of studies

Animal intervention studies (with control group).

#### 2.2.2 Types of animal models

All animal models with cerebral ischemia (all species, all sexes).

#### 2.2.3 Types of interventions

Using 3-MA on in vivo animal model of cerebral ischemia. All timings, frequencies and dosages of treatment are eligible for inclusion.

#### 2.2.4 Types of comparators

Using the same modeling method with the experimental group; vehicle-treated control animals, or sham-treated animals, or animals undergoing no treatment at all.

#### 2.2.5 Types of outcome measures

Primary outcome: infarct volume; neuro-behavioral scores.

Secondary outcome: cerebral blood flow, blood–brain barrier permeability, cerebral hemorrhage, brain water content.

### 2.3 Exclusion criteria

1. Not a primary study, or non-intervention studies;
2. Not cerebral ischemia animal model(s) in vivo; or knockout animal studies; or ex vivo studies, or in vitro studies, or studies in humans or in sillico studies;
3. No treatment with 3-MA, or co-treatments with additional drugs, or using 3-MA to rescue genetic knock-down animals;
4. No control groups, or using additional drugs other than the vehicle in the control group;
5. No relevant outcomes reported, or missing key data (N, SD, SE etc);
6. Conference abstract that does not contain appropriate data for extraction;
7. Studies which will be published later in full.

### 2.4 Search methods for identification of studies

#### 2.4.1 Electronic searches

The following electronic bibliographic databases will be included in the electronic search strategy: PubMed, EMBASE, Scopus, Science Direct, and Web of Science. No publication date or language restrictions will be applied. All retrieval up to August 31, 2020.

The reference lists of included studies will be screened for additional eligible studies not retrieved by our search. The searches will be re-run just before the final analyses to retrieve the most recent studies eligible for inclusion.

#### 2.4.2 Searching strategy

The full search strategy is based on the search components “ischemic stroke”, “animal” and “3-MA”. The search strategy for PubMed is shown in Table 1, and other electronic databases will be searched with the same strategy.

**Table 1:** Search strategy for PubMed database.

Number	Search Terms
#1	“Stroke” [Mesh] OR “Cerebral Infarction” [Mesh] OR “Brain Infarction” [Mesh] OR “Brain Ischemia” [Mesh] OR “Infarction, Anterior Cerebral Artery” [Mesh] OR “Infarction, Middle Cerebral Artery” [Mesh] OR “Infarction, Posterior Cerebral Artery” [Mesh] OR “Hypoxia-Ischemia, Brain” [Mesh] OR “Stroke, Lacunar” [Mesh] OR “Brain Stem Infarctions” [Mesh] OR “Lateral Medullary Syndrome”
#2	Ischemic Stroke [Title/Abstract] OR Ischaemic Stroke [Title/Abstract] OR Cerebral Ischemia [Title/Abstract] OR Cerebral Ischaemia [Title/Abstract] OR Brain Ischaemia [Title/Abstract] OR Ischemic Brain [Title/Abstract] OR Ischaemic Brain [Title/Abstract] OR Cerebral Infarct [Title/Abstract] OR Brain Infarct [Title/Abstract] OR Cerebral Artery Infarct [Title/Abstract] OR Cerebral Artery Infarction [Title/Abstract] OR Cerebral Circulation Infarction [Title/Abstract] OR Cerebral Artery Thrombosis[Title/Abstract] OR Cerebral Artery Thrombotic Infarction [Title/Abstract] OR Cerebral Artery Embolic Infarction [Title/Abstract] OR ACA Infarction [Title/Abstract] OR MCA Infarction [Title/Abstract] OR PCA Infarction [Title/Abstract] OR Cerebral Artery Occlusion [Title/Abstract] MCAO [Title/Abstract] OR tMCAO [Title/Abstract] OR pMCAO [Title/Abstract] OR Brain Venous Infarction [Title/Abstract]
#3	#1 OR #2
#4	3-Methyladenine [All Fields] OR 3-MA[All Fields] OR 3MA [All Fields] OR NSC 66389 [All Fields] OR 3-Methyl-3H-purin-6-amine [All Fields]
#5	“Models, Animal” [Mesh] OR “Rats” [Mesh] OR “Mice” [Mesh] OR “Rabbits” [Mesh]
#6	Animal Disease Model [Title/Abstract] OR Animal Model [Title/Abstract] OR Preclinical [Title/Abstract] OR Rat [Title/Abstract] OR Mice [Title/Abstract] OR Mouse [Title/Abstract] OR Murine [Title/Abstract] OR Hamster [Title/Abstract] OR Guinea Pig [Title/Abstract] OR Gerbil [Title/Abstract] OR Jird [Title/Abstract] OR Chinchilla [Title/Abstract] OR Rodent [Title/Abstract] OR Rabbit [Title/Abstract] OR Dog [Title/Abstract] OR Canine [Title/Abstract] OR Cat [Title/Abstract] OR Feline[Title/Abstract] OR Bovine [Title/Abstract] OR Cattle [Title/Abstract] OR Cow [Title/Abstract] OR Caprine [Title/Abstract] OR Goat [Title/Abstract] OR Ovine [Title/Abstract] OR Sheep [Title/Abstract] OR Equine [Title/Abstract] OR Horse [Title/Abstract] OR Avian [Title/Abstract] OR Chicken [Title/Abstract] OR Poultry [Title/Abstract] OR Porcine [Title/Abstract] OR Pig [Title/Abstract] OR Swine[Title/Abstract] OR Monkey [Title/Abstract] OR Simian [Title/Abstract] OR Macaque [Title/Abstract] OR Primate [Title/Abstract]
#7	#5 OR #6
#8	#3 AND #4 AND #7

### 2.5 Data collection and analysis

#### 2.5.1 Selection of studies

**Figure 1:**
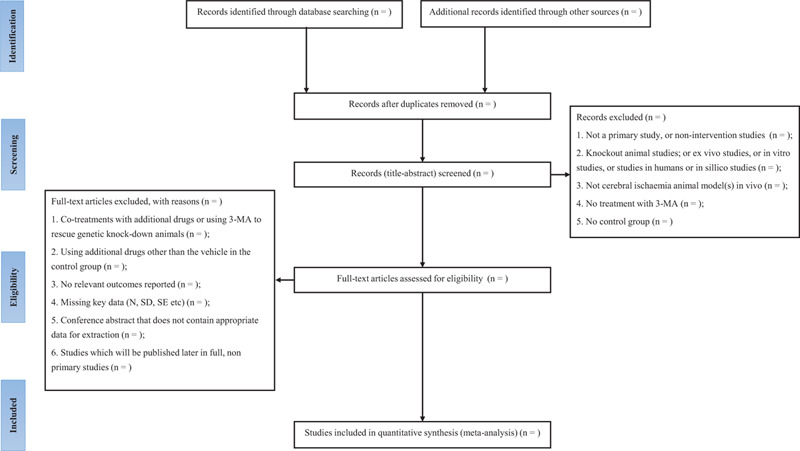
Flow diagram of the study selection process. Adapted from Preferred Reporting Items for Systematic reviews and Meta-Analysis Protocols (PRISMA-P) flow diagram.^[28]^

All reviewers will receive professional training to understand the objective and process of the review before the selection of studies. Literature search results will be imported into ENDNOTE X8 software. The duplicates will be removed. For studies that have been updated, the older one will be excluded, or can be used as supplementary data in further research. Titles and abstracts will be screened independently by 2 reviewers (AZ and ZHZ). Full texts will be obtained for eligible studies and will be screened independently (AZ and ZHZ). Discrepancies will be resolved through discussion, or by consulting a third reviewer (YYS). The procedures of study selection will be performed in accordance with the Preferred Reporting Items for Systematic reviews and Meta-Analysis flow chart (see Fig. 1).^[28]^

#### 2.5.2 Data extraction and management

Two reviewers (AZ and SYJ) will establish a sheet using Microsoft Excel 2010, pilot and refine this form using 10 initial studies. After the form has been developed, the 2 reviewers will extract data from the text and figure/table independently, including:

1. Name of first author, year of publication, language, email address, journal;
2. Animal species, strain, age, weight, and sex of animals used; comorbidities; anesthetics and ventilation;
3. Number of experimental groups and control groups; number of animals per group; methods to establish animal models; methods of ischemia induction and the duration of ischemia; methods of reperfusion induction and the duration of reperfusion;
4. Dose, route, vehicle, frequency, and timing of drug administration;
5. Timing and methods of outcome assessment; the outcomes data of mean value and standard deviation (infarct volume, neuro-behavioral scores, cerebral blood flow, blood-brain barrier permeability, cerebral hemorrhage, and brain water content).

For extraction of dose for each comparison, a standardized dose in μg/kg was calculated based on the dose of 3-MA administered and the number of times that dose was administered. In case an outcome is measured at multiple time points, data from the time point where the efficacy is the highest will be included. Discrepancies will be identified and resolved through discussion (with a third author where necessary), or by consulting a third reviewer (YYS). We first try to extract numerical data from tables, text or figures. If these are not reported, we will extract data from graphs using digital ruler software. In case data is not reported or unclear, we will attempt to contact authors by e-mail (max. 2 attempts).

#### 2.5.3 Assessment of risk of bias and reporting quality of included studies

In this study, quality assessment of the included studies, and risk of bias will be evaluated according to Systematic Review Center for Laboratory Animal Experimentation (SYRCLE) risk of bias tool^[29]^ and the Collaborative Approach to Meta-Analysis and Review of Animal Data from Experimental Studies (CAMARADES) checklist,^[30]^ respectively.

Two investigators (AZ and ZHZ) will independently assess the risk of bias and study quality, after being trained and calibrated to ensure uniformity in the evaluation of the criteria. Differences of opinion that cannot be resolved by discussion will be resolved by invoking a third investigator (YYS).

#### 2.5.4 Measurement of treatment effect and data analysis

Review Manager 5.3 and Stata 15.1 will be used in data analysis. For dichotomous data, a risk ratio with 95% confidence intervals will be used for analysis. For continuous data, a mean difference or a standard mean difference with 95% confidence intervals will be used for analysis.

#### 2.5.5 Assessment of heterogeneity

*I*^2^ statistic will be calculated for quantifying heterogeneity among included studies (*I*^2^ > 50%, means large heterogeneity; 25% < *I*^2^ ≦ 50%, means medium heterogeneity; and 0 ≦ *I*^2^ ≦ 25%, means small heterogeneity), and meta-regression will be used to investigate sources of heterogeneity.

#### 2.5.6 Assessment of publication bias

We plan to implement publication bias when more than 10 studies are included for one outcome. The methods for assessment of publication bias will be: Funnel plot, Egger linear regression and adjusted for trim and fill.

#### 2.5.7 Data synthesis

A meta-analysis will be performed for all outcome measures reported in 10 or more articles. For subgroup analysis a minimum of 5 studies per subgroup is required. If meta-analysis is not possible, data will be reported through a descriptive summary. The statistical model for the analysis will be a random effects model to account for the anticipated heterogeneity between studies that will be included in the review. Where different neurobehavioral outcomes are reported from the same cohort of animals for the same time point, these (pre-nested) comparisons will be combined using fixed-effects meta-analysis (nesting) and this summary estimate will be used in the random-effects model.

#### 2.5.8 Subgroup analysis

If the necessary data are available, subgroup analyses will be done for animals with the stage of ischemia and the stage of reperfusion separately. Within each stage, and overall, we also plan to do a subgroup analysis by the timing of drug administration (before ischemia, after ischemia and before reperfusion, after reperfusion).

The following study characteristics will be examined as potential source of heterogeneity: species/strain (stratified per species/strains); sex (stratified per sex); model of ischemia (stratified per modeling method); duration of ischemia (linear); duration of reperfusion (linear); time after ischemia when outcome was measured (linear); treatment dose (linear); timing of administration (linear); blinding of outcome assessment reported (stratified yes vs no); comorbidities (stratified yes vs no); study quality (linear); randomization to treatment/control (stratified yes vs no); allocation concealment (stratified yes vs no). For stratified analyses, a minimum number of 5 studies per subgroup are required.

#### 2.5.9 Sensitivity analysis

Stratified meta-analysis will be used as an alternative method to investigate sources of heterogeneity. Stratifications will be considered in 2 domains: study design and study quality.

Each domain will be tested at *P* < .05 overall and a Holm Bonferroni adjusted critical *P* value will be calculated to account for the number of parameters tested within each domain. Other sensitivity analyses will be carried out if necessary (e.g., if there are outliers that may skew results the analysis will be re-run excluding any outliers and the results of both analyses reported).

## 3 Discussion

In 1982, 3-MA was first identified as an autophagy inhibitor for its effects on suppressing the formation of autophagosomes and autophagic/lysosomal protein degradation.^[31]^ Recent studies have shown that 3-MA inhibits autophagy by blocking the class III PI3K pathway, which is essential to phagophore formation and the nucleation of the autophagosome.^[32,33]^ Experiments on 3-MA effects on cerebral infarction animal models are really important to the development of novel therapeutic agents to treat ischemic stroke.^[34,35]^ Some scientists found 3-MA improves cell viability and decreases cell death by inhibiting autophagy, so it can protect brain from ischemic injury^[36,37]^; while other studies suggest that 3-MA worsens neuronal death in ischemic stroke by decreasing autophagic flux and blocking the clearance of impaired organelles.^[38,39]^

This study aims to analyze whether inhibiting autophagy by 3-MA brings protection or damage to IP neurons in ischemic stroke; furthermore, it also helps to clarify the specific role of autophagy in cerebral infarction. Our systematic review and meta-analysis can show the advantages and limitations of the current literature in this field; additionally, this research can also preclude unnecessary study replication, improve the development of future clinical trials of autophagy-targeted therapy strategies, and bring more hope to patients with ischemic stroke.

## Author contributions

Ao Zhang designed this research and drafted the manuscript, Xinchang Zhang tested the feasibility of the study, Yangyang Song, Zhihui Zhang, Siyuan Jiang, Siqi Chang, Zhengyun Cai, Furong Liu contributed to the development of the selection criteria, and the risk of bias assessment strategy. Guangxia Ni read, provided feedback and approved the final manuscript. All authors approved the final version of the manuscript.

**Conceptualization:** Zhengyun Cai.

**Investigation:** Xinchang Zhang, Zhihui Zhang.

**Methodology:** Yangyang Song, Siyuan Jiang.

**Resources:** Zhengyun Cai, Furong Liu.

**Software:** Siqi Chang.

**Supervision:** Guangxia Ni.

**Validation:** Xinchang Zhang.

**Writing – original draft:** Ao Zhang.

## Abbreviations

Abbreviations: 3-MA = 3-Methyladenine, IP = ischemic penumbra.
